# Effect of Video Camera Angle on the Detection of Compensatory Movements during Motion Observation

**DOI:** 10.3390/life13122250

**Published:** 2023-11-23

**Authors:** Norio Kato, Yuki Fujino

**Affiliations:** 1 Department of Physical Therapy, Faculty of Health Sciences, Hokkaido University of Science, Sapporo 006-8585, Japan; 2 Division of Rehabilitation Sciences, Graduated School of Health Sciences, Hokkaido University of Science, Sapporo 006-8585, Japan; 9233201@hus.ac.jp

**Keywords:** motion observation, compensatory movement, shooting angle

## Abstract

When exercise instructions are provided over the Internet, such as in online personal training, an instructor checks the user’s form by watching their motion video recorded using a single camera device. However, fixed shooting angles may affect the detection of incorrect forms, including compensatory movements. This study aimed to verify whether differences in the shooting direction could influence compensatory movement detection by conducting motion observation using training motion videos shot from two angles. Videos of four training movements, including compensatory movements, were simultaneously captured from the front and side. Ten university students studying physical therapy watched the videos from each angle to detect compensatory movements. This study revealed significant differences between the plane of motion in which the compensatory action occurred and the direction of shooting for the false responses in the compensatory action detection for the three movements (*p* < 0.05). The results indicated that the shooting direction and the plane of motion in which the compensatory action occurred affected the detection of compensatory movements, which was attributable to differences in information on the amount of joint change depending on the direction of joint motion observation and to a lack of binocular visual information necessary for depth motion detection.

## 1. Introduction

With the spread of digital infrastructure, various services and personal training programs are currently provided over the Internet [1,2]. In the area of healthcare, extensive research and the development of equipment have been performed for online medical care and telerehabilitation [3,4]. In addition, the recent coronavirus disease 2019 pandemic has restricted people’s activities [5,6], and its impact on the health of both athletes and citizens has been highlighted.

Several studies have reported the effectiveness of Internet-based healthcare services and have shown that telerehabilitation is as effective as conventional rehabilitation for a wide range of diseases, including cerebrovascular diseases (e.g., cerebral infarction), cardiac diseases (e.g., myocardial infarction), respiratory diseases (e.g., chronic obstructive pulmonary disease), and musculoskeletal diseases (e.g., knee osteoarthritis) [7,8,9,10]. Furthermore, several studies on online training have demonstrated its effectiveness. In particular, Kikuchi et al. compared improvements in fitness parameters after training between a face-to-face exercise instruction group and an online training group using Zoom and observed no differences between these groups [11]. Bulguroglu et al. also found no differences in effectiveness between online and face-to-face Pilates exercises [12], thereby validating the online training methodology. Daveri et al. investigated the effectiveness of online training in a supervised group, a group using only videos, and a group using only handouts in training programs and concluded that supervised training was the most effective method [13]. Arslan et al. conducted online training for women with patellofemoral pain syndrome and reported that the group provided with direct instructions experienced less pain and fear during the activity [14]. These findings suggest that online personal training can be an alternative to traditional face-to-face training and that the provision of direct instructions under the supervision of a trainer is more effective than on-demand training. Additionally, online healthcare services are not only effective for exercises but also cost-effective because they eliminate time and location constraints [15].

The ability to make detailed observations of service users’ movements is regarded as one of the most important factors when providing exercise instructions via the Internet. Accordingly, online healthcare services utilize online conferencing platforms, sensors such as depth cameras and inertial measurement unit (IMU) sensors, and posture-estimation artificial intelligence (AI) to observe the movement of service users [11,16,17,18,19]. The easiest way to utilize these data is through a videoconferencing system with a webcam. In such a case, the instructor observes the motion video using a web camera and provides instructions for correction if the motion is undesirable. For instance, compensatory movements are produced when motor functions such as muscle strength and joint range of motion (ROM) deteriorate because of aging, as is the case with elderly people. However, compensatory movements—defined as movements differing from normal movements that are produced to compensate for the reduced functions of movements of other muscles and joints [20]—should be avoided when motor function recovery is possible. In rehabilitation, compensatory movements improve function in the short term but reduce the effectiveness of rehabilitation and hinder motor function recovery in the long term [21]. Additionally, compensatory movements produced during strength training also reduce the load on the originally targeted muscle, which not only decreases the effectiveness of training but also leads to injury if not performed correctly [22]. Therefore, observing users’ movements and detecting compensatory movements during exercise are important.

When providing face-to-face instructions in hospitals and training facilities, the instructor often detects compensatory movements by observing the user’s movements from various angles. Compensatory movements can also be detected using IMU sensors that can assess acceleration, angular velocity, and geomagnetism [23,24,25,26,27]. Detecting compensatory movements using measurement devices has the advantage of being easy because the movements of each joint are quantitatively measured. Nevertheless, in online training, movements are often captured using only one smartphone or tablet camera, making it impossible to quantify the movements of each joint and observe them from multiple perspectives.

MacKenzie et al. studied movement observations and compared eye movements between a group of occupational therapists and a group with no training in human movement or health while observing videos of stroke survivors walking, performing household chores, or using the toilet [28]. While the point of observation has been shown to differ depending on the presence or absence of expertise, these videos were shot from a fixed angle, and the observation did not address problems with movements, which may be both considered points for improvement. Hickey et al. observed video recordings of shoulder flexion and abduction movements from the back to determine whether shoulder symptoms and their types could possibly be classified [29]. They reported that even experienced physical therapists experienced difficulty in judging symptoms; however, the effects of changing the angle of video recordings on movement observations were not examined because the videos were taken only from the back.

The present study aimed to investigate the influence of overlooking compensatory movements by conducting motion observation using videos of the same motion taken from two different angles. If the results of this study indicate a relationship between a single fixed-camera video and missing compensatory movements, it can be shown that devising new camera angles or combining them with other methods is necessary.

## 2. Materials and Methods

We used a quantitative research design to investigate the effects of the shooting direction in which movements are captured during the detection of compensatory movements. The details of this process are described below.

### 2.1. Participants

A total of 10 healthy adults (8 men and 2 women; mean age: 21.6 ± 0.51 years) were included as study participants. As the experimental task required expertise in observing human movements, the participants were limited to those who had studied anatomy and kinesiology at a training school for physical therapists and had experience in movement observation and movement analysis during exercises and practical training. Participants with abnormal visual function were excluded. All study protocols conformed to the 1964 Declaration of Helsinki and were approved by the Institutional Review Board of Hokkaido University of Science (approval no. 645). Informed consent was obtained from all participants.

### 2.2. Motion Observation Assignment Video

**Figure 1:**
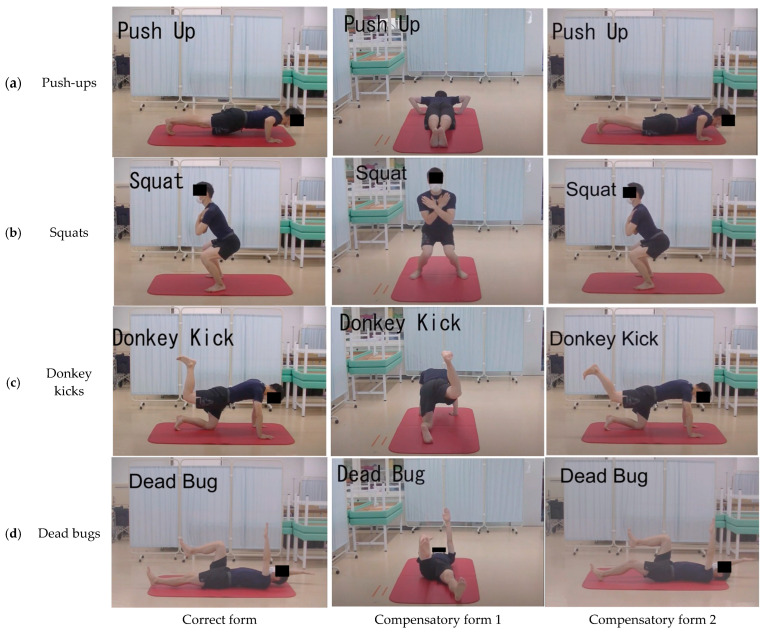
Forms of training movements used in the experiment. (**a**) push-ups, (**b**) squats, (**c**) donkey kicks, and (**d**) dead bugs.

In this study, four types of bodyweight training movements were used as target movements for movement observation—namely, push-ups, squats, donkey kicks, and dead bugs (Figure 1). Each training exercise was performed by a university student enrolled in a training school for physical therapists who received instructions from a physical therapist. Training movements were simultaneously filmed in two directions using a video camera (HC-X920M, Panasonic Inc., Tokyo, Japan) mounted on a tripod. The resolution of the video camera was set at 1920 × 1080 pixels. One of the shooting directions was the same for all movements, with the video camera positioned laterally to capture videos of motion in the sagittal plane. The video camera was positioned in front of squats and caudally for other training movements to record videos in the frontal and horizontal planes. The position of the video camera was adjusted so that a full-body image was captured during training.

Each training movement was performed under three conditions: the correct form and two incorrect forms that included compensatory movements. The correct form of each training movement was obtained from books on training [30,31]. Additionally, two types of compensatory movements were used: one in the sagittal plane and the other in the frontal or horizontal plane. The correct form of each of the four training movements and the definitions of the two compensatory movements are provided below.

#### 2.2.1. Push-Ups (Figure 1a)

Correct form: The hands were slightly wider than shoulder-width apart, the feet were shoulder-width apart on the floor, and the heels to the feet were in a straight line. The arms were placed immediately below the elbows, with the sides of the arms open at 45°. The gluteus and abdominal muscles were contracted, and the entire body was tightened and lowered until the chest touched the floor. The motion was reversed, and the body was pushed up until the elbows were fully extended.

Compensatory form 1 (frontal plane): Compensation by the pectoralis major muscle was performed by executing the form with the shoulder joint abducted at >45°.

Compensatory form 2 (sagittal plane): The lumbar spine was extended when lowering the body to compensate for the reduced pushing distance.

#### 2.2.2. Squats (Figure 1b)

Correct form: The feet were hip-width apart, with toes pointing slightly outward. The upper limbs were folded in front of the chest, and both shoulder joints were held in a 90° flexed position. When lowering the body, the upper body should be parallel to the lower leg, whereas the thigh should be parallel to the floor. The knees were not extended far from the toes. The motion was reversed, returning to the starting position.

Compensatory form 1 (frontal plane): When lowering the body, the knee was brought inward via internal rotation of the hip joint, thereby reducing the load on the quadriceps.

Compensatory form 2 (sagittal plane): When lowering the body, the load on the quadriceps was reduced by extending the trunk.

#### 2.2.3. Bent-Leg Donkey Kick (Figure 1c)

Correct form: On all fours, the head, neck, and spine were in the neutral position, with the hands directly under the shoulders and the knees directly under the hip joints. One leg was raised backward while maintaining the knee joint at 90° flexion. The motion was reversed, returning to the starting position.

Compensatory form 1 (frontal plane): The hip joint was abducted when raising one leg, thereby reducing the load on the gluteus maximus muscle.

Compensatory form 2 (sagittal plane): Compensation with the hamstrings was executed by extending the knee joint when raising one leg backward.

#### 2.2.4. Dead Bug (Figure 1d)

Correct form: The participants lay on their backs, with shoulders, hips, and knees flexed at 90°. One lower limb and the opposite upper limb were extended toward the floor. The upper and lower limbs were stopped by lightly touching the floor. The lumbar spine was maintained in a neutral position. The motion was reversed, returning to the starting position. After returning to the starting position, the same movement was performed for the other upper and lower limbs.

Compensatory form 1 (frontal plane): When lowering one leg, the hip joint was abducted to compensate for the load on the tensor fascia femoris and suture muscles.

Compensatory form 2 (sagittal plane): When lowering one leg, stopping before the knee joint was fully extended reduced the load on the trunk and hip flexor muscles.

One optimal trial was cut from each training movement field, and six trials were repeated as in the experimental video. A physical therapist checked the videos and confirmed that the three form sets for each task movement were in accordance with the conditions.

### 2.3. Experimental Procedure

Experiments were conducted in a quiet room. Chairs and a table were placed in front of a 50-inch display (ILD-B50UHDS-B, IRIS OHYAMA Inc., Sendai, Japan) mounted on a display stand, and two participants performed the task simultaneously. The height from the floor to the lower edge of the display was 1.5 m, and the distance from the display screen to the table was 2 m. Prior to the start of the task, the participants were provided with an explanation of the experimental procedure and the correct form of the four training movements using videos and handouts.

The participants completed a questionnaire regarding their awareness of the four training movements, their knowledge of the form of each movement, and whether they had ever taught them. The questionnaire was created using Google Forms, and the participants answered the questionnaire on their own smartphones or PCs. After completing the questionnaire, the participants performed a motion observation task. All videos used in the experiment included compensatory movements. Two videos were prepared for each compensatory action, with one for each shooting direction, generating a total of 16 task videos. The participants were not told that any videos contained compensatory movements. After completing the observation of one video, the participants responded whether compensatory movements were present or absent for the observed actions using Google Forms. Responses were entered in the same way as for the questionnaire. The response time was 2 min. If compensatory movements were observed, the participants were asked to describe the joints in which compensatory movements occurred and the direction of the movements. The videos were played in random order, and the above procedure was repeated until the participants completed the entire task.

### 2.4. Data Analysis

A physical therapist reviewed the participants’ responses to the observation task and made judgments based on their responses. Responses pertaining to compensatory movements were summarized in a 2 × 2 contingency table and were divided into three categories—namely, knowledge of movements, teaching history, and shooting direction. Additionally, only erroneous answers regarding compensatory movements were extracted and summarized in the 2 × 2 contingency table regarding the shooting direction and plane of motion in which the compensatory movements occurred. Data were analyzed using the chi-square test of independence or Fisher’s exact probability test if the expected frequency was less than 5 in >20% of the cells. Statistical analyses were performed using SPSS version 28 (IBM Corp., Chicago, IL, USA), with the significance level set at <5%.

An a priori power analysis was performed using the χ^2^-test model of G*power 3.1. An adequate sample size was estimated to be 88 based on a power of 0.3 and an alpha of 0.05. However, the required sample size was not reached in this study.

## 3. Results

Table 1 presents the results regarding the knowledge of the form of each training movement and the correctness of compensatory movements. The results of Fisher’s exact probability test indicated no significant differences between the three movements, except for squats (push-up: *p* = 0.608; donkey kick: *p* = 0.243; dead bug: *p* = 0.207). Squats were excluded from the analysis because none of the participants had knowledge of the correct form.

Table 2 presents the results regarding the teaching experience and correctness of compensatory movements for each training movement. Except for the donkey kick, no significant differences were detected between the three movements (push-up: χ^2^(1) = 0.114, *p* = 0.736; squat: *p* = 0.562 per Fisher’s exact test; dead bug: *p* = 0.408 per Fisher’s exact test). The donkey kick was excluded from the analysis because none of the participants had taught it before.

Table 3 presents the results regarding the shooting direction and correctness of compensatory movements. The results of the chi-square test indicated no significant differences between the four types of movements (push-up: χ2(1) = 2.849, *p* = 0.091; squat: χ^2^(1) = 0.440, *p* = 0.507; donkey kick: χ^2^(1) = 1.129, *p* = 0.288; dead bug: χ^2^(1) = 2. 849, *p* = 0.091).

Only wrong answers regarding compensatory movements were extracted (Table 4) and statistically analyzed with Fisher’s exact test with respect to the shooting direction and motor aspect of compensatory movements. Except for push-ups, significant differences were found for the three training movements (push-up: *p* = 0.343; squat: *p* < 0.001, φ = 0.930; donkey kick: *p* = 0.024, φ = 0.810; dead bug: *p* = 0.021, φ = 0.720). Residual analysis showed that compensatory movements in the frontal or horizontal plane were more frequently missed when the shooting direction was lateral, whereas compensatory movements in the sagittal plane were more frequently missed when the shooting direction was frontal or from the tail.

## 4. Discussion

The present study investigated whether the shooting angle could influence the detection of undesirable movements, including compensatory movements, when checking exercise forms using video images captured from two different directions. The results indicated no significant relationship between the knowledge of training movements, teaching experience, or compensatory movement detection rate and also showed no significant relationship between the shooting direction or compensatory movement detection. Conversely, when focusing on the error method of compensatory movement detection, a significant relationship was found between the shooting direction and the motion plane in which compensatory movements occurred. These results suggest that movement observation using videos captured by a single camera may miss the appearance of compensatory movements, even if observers possess expert knowledge of the exercise. One possible reason for this oversight is that the amount of information on these movements is limited when a video is captured from a fixed camera angle.

Compensatory movements refer to the emergence or substitution of a new movement pattern resulting from the adaptation of the remaining motor elements [20]. In other words, when performing a task movement in an area with impaired physical function, an area that should not normally participate in the movement compensates for the impaired function. For example, if upper limb motion is limited owing to a functional decline during reaching, trunk flexion and rotation can be utilized to execute the task movement [32]. If the strength of the iliopsoas muscle, which is the main action muscle for hip flexion, is reduced, hip flexion may be accompanied by abduction and external rotational movements owing to the compensatory action of the suture muscles [33]. Thus, because compensatory movements produce muscle activities and joint movements that differ from normal movements, the detection of movements in different planes is important when observing movements. To avoid overlooking the movements that occur in different planes of motion, physical and occupational therapists must observe them from various viewpoints when analyzing human movements, as there exist differences in the information regarding the amount of change in a joint depending on the direction in which the joint motion is observed. For instance, consider the amount of foot change in space when the hip joint of a person with a lower limb length of 70.0 cm is abducted at 5°. If the motion is observed in the frontal plane, the change in the horizontal direction of the foot would be 6.1 cm. By contrast, when the motion is observed in the sagittal plane, the vertical change would be 0.27 cm. Detecting spatial coordinate changes in the distal parts of segments, such as the upper and lower limbs, in addition to angular changes in the joints, is considered to be easiest when the axis of motion is perpendicular to the plane of motion being observed (i.e., when the direction of the line of sight and the direction of the axis of motion coincide).

Another possible explanation for missed compensatory movements is the influence of the visual detection process on motion in the depth direction, which may be related to the shooting direction and plane of motion in which compensatory movements occur. When humans detect their own motion or that of surrounding objects, they integrate various sensory inputs, such as vision, hearing, vestibular sensation, and touch; this phenomenon is known as motion perception. When detecting the motion of an object by looking at a video image, as in this study, the sensory input source is vision. Visual motion perception is known as motion vision. When the vision obtains information from the outside world, changes in light projected onto the retina are detected by photoreceptor cells. In motion perception, the spatiotemporal changes in luminance projected onto the retina are captured and processed in the primary visual cortex, the MT cortex, and the MST cortex, which mainly detect the translational motion of objects in the frontal plane [34]. However, object motion in 3D space is not limited to motion in the plane perpendicular to the observer’s line of sight, that is, vertical and horizontal, but also includes anteroposterior motion parallel to the observer’s line of sight, that is, motion in the depth direction. Binocular disparity and convergence are involved in the detection of this anteroposterior shift. Rockers et al. showed that the MT complex (MT+) encodes differences in disparity and retinal velocity from binocular visual information and pointed out its involvement in sensitivity to 3D motion [35]. In this study, because the task images were presented on a display, changes in the anteroposterior direction were recognized by the expansion and contraction of the distal segments of the upper and lower limbs. In this case, physiological depth information, such as binocular disparity, was not added, which may have made it difficult to detect changes in the anteroposterior direction. In addition to information on changes in luminance and contrast projected onto the retina, the detection mechanism of motion by motion vision is affected by subtler factors, such as the texture of the object, contrast reversal, and velocity of motion [36]. Therefore, it is necessary to examine the effects of clothing and room brightness in future studies.

The results showed that knowledge of target movements and teaching experience had no effect on overlooking compensatory movements. This was due to the fact that all participants had knowledge of physical therapy, including anatomy and kinesiology, as well as experience in movement observation and movement analysis. McGinly et al. reported no significant differences in the accuracy of detecting abnormal movements in the gait observation of patients with stroke, depending on the number of years of experience [37]. They also speculated that years of experience might be a factor in the decision-making stage when analyzing movements based on the information obtained. By contrast, MacKenzie et al. examined the differences in gaze between occupational therapists and non-medical groups when asked to observe the same movement [28]. The results showed differences in the gazing points between the two groups for movements that were unfamiliar in daily life. The participants in this study were university students studying physical therapy, and all of them had knowledge of exercise; therefore, it is thought that they were able to appropriately visualize normal movements, even for movements that were not well known. In addition, it was possible to infer compensatory movements that occur when motor functions such as muscle strength and joint ROM are impaired, and it is predicted that it will be possible to focus on these points during movement observation. To verify this, it is necessary to measure the line of sight during the movement observation. Furthermore, it was not possible to accurately determine the extent to which participants had knowledge of the correct exercise form. In the future, it will be necessary to ask participants to explain or demonstrate the correct movement form before the experiment.

This study has some limitations. First, the study included a small number of study participants who were only pre-licensed physical therapy students. Students and licensed physical therapists have different perspectives on this subject. Second, the task movements were limited. For training movements that are also used in rehabilitation or daily life, it is possible to reduce the influence of missing compensatory movements based on the observers’ knowledge and experience. Third, compensatory movements were performed by healthy participants whose physical function was not impaired. As the magnitude of compensatory movements varies depending on the degree of decline in physical function, future validation using exercise videos of people with various types of physical function impairments is required. Fourth, the shooting angle was limited to two directions. It is necessary to verify the videos from various angles, such as from a bird’s-eye view or in an oblique downward or upward direction. Finally, the video display devices and resolutions were limited to one type, and images with a resolution of 1980 × 1024 pixels were displayed on a 50-inch display. Nevertheless, it is expected that the detection of compensatory actions will be affected by lower and higher resolutions and by the size of devices used to view the actions. Future studies should compare multiple resolutions and display sizes.

## 5. Conclusions

The present study examined the effect of video camera shooting angles during exercise on the detection of compensatory movements, assuming online personal training and telerehabilitation, and revealed that the detection of compensatory movements was affected by the shooting direction and the plane of motion in which compensatory movements occurred. To improve the detection accuracy of compensatory movements, we recommend using video images taken from multiple angles and using motion measurement devices such as IMU sensors.

## Figures and Tables

**Table 1:** Results of compensatory movement detection based on the knowledge of the form of training movements.

	Knowledge	Answer		Fisher’s Exact Test	Effect Size
		Correct	Wrong	*p*-Value	Φ
Push-ups	〇	24	12	0.608	0.053
	×	3	1		
Squats	〇	26	14		
	×	0	0		
Donkey kick	〇	9	3	0.243	0.183
	×	25	3		
Dead bug	〇	10	2	0.207	−0.190
	×	18	10		

〇: The participants were knowledgeable about the correct exercise form. ×: The participants did not have any knowledge of the correct form. Squats were excluded from the analysis because none of the participants had any knowledge of the correct form.

**Table 2:** Results of compensatory movement detection based on experience in teaching training movements.

	Teaching Experience	Answer		Pearson’s Chi-Square Test		Fisher’s Exact Test	Effect Size
		Correct	Wrong	χ^2^ Value	*p*-Value	*p*-Value	Cramer’s V|Φ
Push-up	〇	13	7	0.114	0.736		0.053
	×	14	6				
Squat	〇	23	13			0.562	0.070
	×	3	1				
Donkey kick	〇	0	0				
	×	34	6				
Dead bug	〇	7	1			0.408	−0.191
	×	21	11				

〇: The participants had experience in teaching training movements. ×: The participants had no experience in teaching training movements. The donkey kick was excluded from the analysis because none of the participants had experience in teaching it.

**Table 3:** Results of compensatory movement detection according to the shooting direction.

	Shooting Direction	Answer		Pearson’s Chi-Square Test		Effect Size
		Correct	Wrong	χ^2^ Value	*p*-Value	Cramer’s V
Push-up	Lateral	16	4	2.849	0.091	0.267
	Cranial	11	9			
Squat	Lateral	14	6	0.440	0.507	0.105
	Front	12	8			
Donkey kick	Lateral	13	7	1.129	0.288	0.168
	Caudal	16	4			
Dead bug	Lateral	11	9	2.849	0.091	0.267
	Caudal	16	4			

**Table 4:** Misinterpretation of compensatory movements.

	Shooting Direction	Number of Compensatory Movements Missed		Fisher’s Exact Test	Effect Size
		On the Sagittal Plane	On the Frontal or Horizontal Plane	*p*-Value	Φ
Push-up	Lateral	1	3	0.343	−0.283
	(residual)	(−1.0)	(1.0)		
	Cranial	5	4		
	(residual)	(1.0)	(−1.0)		
Squat	Lateral	1	5	<0.001	−0.930
	(residual)	(−6.2)	(6.2)		
	Frontal	8	0		
	(residual)	(6.2)	(−6.2)		
Donkey kick	Lateral	0	7	0.024	−0.810
	(residual)	(−2.6)	(2.6)		
	Caudal	3	1		
	(residual)	(2.6)	(−2.6)		
Dead bug	Lateral	2	7	0.021	−0.720
	(residual)	(−2.7)	(2.7)		
	Caudal	4	0		
	(residual)	(2.7)	(−2.7)		

With respect to the misinterpretation of compensatory movements, we classified the shooting direction and kinematic aspect of compensatory movements.

## Data Availability

The data presented in this study are available in this article.

## References

[1] McIlroy B., Passfield L., Holmberg H.C., Sperlich B. (2021). Virtual Training of Endurance Cycling—A Summary of Strengths, Weaknesses, Opportunities and Threats. Front. Sports Act. Living.

[2] Galway S.C., Laird M.H.D., Dagenais M., Gammage K.L. (2023). Navigating a New Normal: Perceptions and Experiences of an Online Exercise Program for Older Adults During COVID-19. J. Aging Phys. Act..

[3] Ekeland A.G., Bowes A., Flottorp S. (2010). Effectiveness of telemedicine: A systematic review of reviews. Int. J. Med. Inform..

[4] Suso-Marti L., La Touche R., Herranz-Gomez A., Angulo-Diaz-Parreno S., Paris-Alemany A., Cuenca-Martinez F. (2021). Effectiveness of Telerehabilitation in Physical Therapist Practice: An Umbrella and Mapping Review with Meta-Meta-Analysis. Phys. Ther..

[5] Ammar A., Brach M., Trabelsi K., Chtourou H., Boukhris O., Masmoudi L., Bouaziz B., Bentlage E., How D., Ahmed M. (2020). Effects of COVID-19 Home Confinement on Eating Behaviour and Physical Activity: Results of the ECLB-COVID19 International Online Survey. Nutrients.

[6] Iff S., Frohlich S., Halioua R., Imboden C., Sporri J., Scherr J., Butzke I., Seifritz E., Claussen M.C. (2022). Training Patterns and Mental Health of Bodybuilders and Fitness Athletes During the First Lockdown of the COVID-19 Pandemic-A Cross-Sectional Study. Front. Sports Act. Living.

[7] Tchero H., Tabue Teguo M., Lannuzel A., Rusch E. (2018). Telerehabilitation for Stroke Survivors: Systematic Review and Meta-Analysis. J. Med. Internet Res..

[8] Nacarato D., Sardeli A.V., Mariano L.O., Chacon-Mikahil M.P.T. (2022). Cardiovascular telerehabilitation improves functional capacity, cardiorespiratory fitness and quality of life in older adults: A systematic review and meta-analysis. J. Telemed. Telecare.

[9] Hansen H., Bieler T., Beyer N., Kallemose T., Wilcke J.T., Ostergaard L.M., Frost Andeassen H., Martinez G., Lavesen M., Frolich A. (2020). Supervised pulmonary tele-rehabilitation versus pulmonary rehabilitation in severe COPD: A randomised multicentre trial. Thorax.

[10] Cottrell M.A., Galea O.A., O’Leary S.P., Hill A.J., Russell T.G. (2017). Real-time telerehabilitation for the treatment of musculoskeletal conditions is effective and comparable to standard practice: A systematic review and meta-analysis. Clin. Rehabil..

[11] Kikuchi N., Mochizuki Y., Kozuma A., Inoguchi T., Saito M., Deguchi M., Homma H., Ogawa M., Hashimoto Y., Nakazato K. (2022). The Effect of Online Low-intensity Exercise Training on Fitness and Cardiovascular Parameters. Int. J. Sports Med..

[12] Bulguroglu H.I., Bulguroglu M. (2023). The effects of online pilates and face-to-face pilates in healthy individuals during the COVID-19 pandemic: A randomized controlled study. BMC Sports Sci. Med. Rehabil..

[13] Daveri M., Fusco A., Cortis C., Mascherini G. (2022). Effectiveness of Different Modalities of Remote Online Training in Young Healthy Males. Sports.

[14] Arslan T., Gultekin M.Z. (2023). The effect of a supervised online group exercise program on symptoms associated with patellofemoral pain syndrome in women. Technol. Health Care.

[15] Llorens R., Noe E., Colomer C., Alcaniz M. (2015). Effectiveness, usability, and cost-benefit of a virtual reality-based telerehabilitation program for balance recovery after stroke: A randomized controlled trial. Arch. Phys. Med. Rehabil..

[16] Parker K., Uddin R., Ridgers N.D., Brown H., Veitch J., Salmon J., Timperio A., Sahlqvist S., Cassar S., Toffoletti K. (2021). The Use of Digital Platforms for Adults’ and Adolescents’ Physical Activity During the COVID-19 Pandemic (Our Life at Home): Survey Study. J. Med. Internet Res..

[17] Bell K.M., Onyeukwu C., McClincy M.P., Allen M., Bechard L., Mukherjee A., Hartman R.A., Smith C., Lynch A.D., Irrgang J.J. (2019). Verification of a Portable Motion Tracking System for Remote Management of Physical Rehabilitation of the Knee. Sensors.

[18] Anton D., Berges I., Bermudez J., Goni A., Illarramendi A. (2018). A Telerehabilitation System for the Selection, Evaluation and Remote Management of Therapies. Sensors.

[19] Joo S.Y., Lee C.B., Joo N.Y., Kim C.R. (2021). Feasibility and Effectiveness of a Motion Tracking-Based Online Fitness Program for Office Workers. Healthcare.

[20] Levin M.F., Kleim J.A., Wolf S.L. (2009). What do motor “recovery” and “compensation” mean in patients following stroke?. Neurorehabilit. Neural Repair..

[21] Cirstea M.C., Levin M.F. (2000). Compensatory strategies for reaching in stroke. Brain.

[22] Faigenbaum A.D., Myer G.D. (2010). Resistance training among young athletes: Safety, efficacy and injury prevention effects. Br. J. Sports Med..

[23] Alankus G., Kelleher C. Reducing compensatory motions in video games for stroke rehabilitation. Proceedings of the CHI ‘12: Proceedings of the SIGCHI Conference on Human Factors in Computing Systems.

[24] Chen X., Shao Y., Zou L., Tang S., Lai Z., Sun X., Xie F., Xie L., Luo J., Hu D. (2023). Compensatory movement detection by using near-infrared spectroscopy technology based on signal improvement method. Front. Neurosci..

[25] Dunne A., Do-Lenh S., Gearóid Ó.L., Shen C., Bonato P. (2010). Upper extremity rehabilitation of children with cerebral palsy using accelerometer feedback on a multitouch display. Annu. Int. Conf. IEEE Eng. Med. Biol. Soc..

[26] Delbressine F., Timmermans A., Beursgens L., de Jong M., van Dam A., Verweij D., Janssen M., Markopoulos P. (2012). Motivating arm-hand use for stroke patients by serious games. Annu. Int. Conf. IEEE Eng. Med. Biol. Soc..

[27] Leirós-Rodríguez R., Romo-Pérez V., García-Soidán J.L., Soto-Rodríguez A. (2020). Identification of Body Balance Deterioration of Gait in Women Using Accelerometers. Sustainability.

[28] MacKenzie D.E., Westwood D.A. (2013). Observation patterns of dynamic occupational performance. Can. J. Occup. Ther..

[29] Hickey B.W., Milosavljevic S., Bell M.L., Milburn P.D. (2007). Accuracy and reliability of observational motion analysis in identifying shoulder symptoms. Man. Ther..

[30] Contreras B. (2013). Bodyweight Strength Training Anatomy.

[31] Cook G. (2011). Movement: Functional Movement Systems: Screening, Assessment, Corrective Strategies.

[32] Levin M.F., Liebermann D.G., Parmet Y., Berman S. (2016). Compensatory Versus Noncompensatory Shoulder Movements Used for Reaching in Stroke. Neurorehabilit. Neural Repair..

[33] Brown M.A. (2018). Daniels and Worthingham’s Muscle Testing.

[34] DeYoe E.A., Van Essen D.C. (1988). Concurrent processing streams in monkey visual cortex. Trends Neurosci..

[35] Rokers B., Cormack L.K., Huk A.C. (2009). Disparity- and velocity-based signals for three-dimensional motion perception in human MT+. Nat. Neurosci..

[36] Barton J.J.S. (2021). Motion perception and its disorders. Handb. Clin. Neurol..

[37] McGinley J.L., Goldie P.A., Greenwood K.M., Olney S.J. (2003). Accuracy and reliability of observational gait analysis data: Judgments of push-off in gait after stroke. Phys. Ther..

